# Unilateral and segmental distribution of facial erythema: is it a real port-wine stain?

**DOI:** 10.1186/s41065-020-00143-z

**Published:** 2020-07-07

**Authors:** Qingqing Cen, Yi Sun, Xiaojing Zeng, Yun Liu, Fatao Liu, Hui Chen, Xiaoxi Lin, Ren Cai

**Affiliations:** 1 Department of Plastic and Reconstructive Surgery, Shanghai 9th People’s Hospital, Shanghai Jiao Tong University School of Medicine, Shanghai, PR China; 2 Bio-X Institute, Shanghai Jiao Tong University, Shanghai, PR China; 3 Institutes of Biomedical Sciences, Fudan University, Shanghai, PR China; 4 Department of General Surgery, Xinhua Hospital Affiliated to Shanghai Jiao Tong University School of Medicine, Shanghai, PR China; 5 Institute of Biliary Tract Disease, Shanghai Jiao Tong University School of Medicine, Shanghai, PR China

**Keywords:** EPHB4 mutation, Capillary malformation-arteriovenous malformation, Port-wine stain, Erythema

## Abstract

Capillary malformation-arteriovenous malformations (CM-AVMs) caused by a RASA-1 or EPHB4 mutation are characterized as hereditary sporadic or multifocal capillary malformations (CMs), associated with potential fast-flow vascular anomalies underlying erythema lesions. Because of the similar phenotype, CM-AVMs should be considered in the differential diagnosis of isolated CMs as well as other disorders with an erythema phenotype, such as hereditary hemorrhagic telangiectasia (HHT).

Herein, we report a male patient with facial erythema. Red lesions were located in the V1 region of his left face, the V2 and V3 regions on his right side, and the nasal back. The patient was initially thought to have PWSs because of the unilateral and segmental distribution of his red facial lesions. In contrast to a previous diagnosis, we diagnosed the child with capillary malformation-arteriovenous malformation type 2 (CM-AVM2) based on a family history of erythema, the results of physical examination and ultrasound raising potential fast-flow lesions, and a genetic study revealing a germline EPHB4 mutation. This study emphasizes the importance of differential diagnosis for PWS and CM-AVM. A single clinical diagnosis can be limited, and molecular diagnosis is recommended to provide more information for the evaluation of the potential risk of fast-flow lesions underlying erythema lesions if necessary.

## Background

Capillary malformations (CMs), also known as port-wine stains (PWSs), are the most common slow-flow vascular malformation in the skin, affecting approximately 0.3% of newborns [1, 2]. CMs can present as erythema with pink or red color in the skin and cutaneous tissue that progressively grows with the growth of individuals and does not regress spontaneously [3]. CMs can be isolated or can occur as a component of capillary malformation–arteriovenous malformation (CM-AVM) syndrome associated with arteriovenous malformation (AVM) or arteriovenous fistula (AVF) [4, 5]. CMs characteristic of this syndrome are smaller and pinker compared with isolated CMs, and many exhibit a surrounding pale or blanched halo [6]. CM-AVMs, are an autosomal dominant disorder caused by germline RASA-1 mutations or EPHB4 mutations [4, 7, 8]. Because of the similar phenotypes, CM-AVMs should be considered in the differential diagnosis of isolated CMs as well as other disorders with erythema phenotypes, such as hereditary hemorrhagic telangiectasia (HHT) [9].

Herein, we report one male patient with facial erythema who was initially thought to have PWSs because of the unilateral and segmental distribution of his red facial lesions but was later diagnosed with CM-AVM2 syndrome based on a germline EPHB4 mutation. We also describe the main clinical manifestations, imaging findings and molecular information that differentiate PWS and CM-AVM.

## Case presentation

**Fig. 1:**
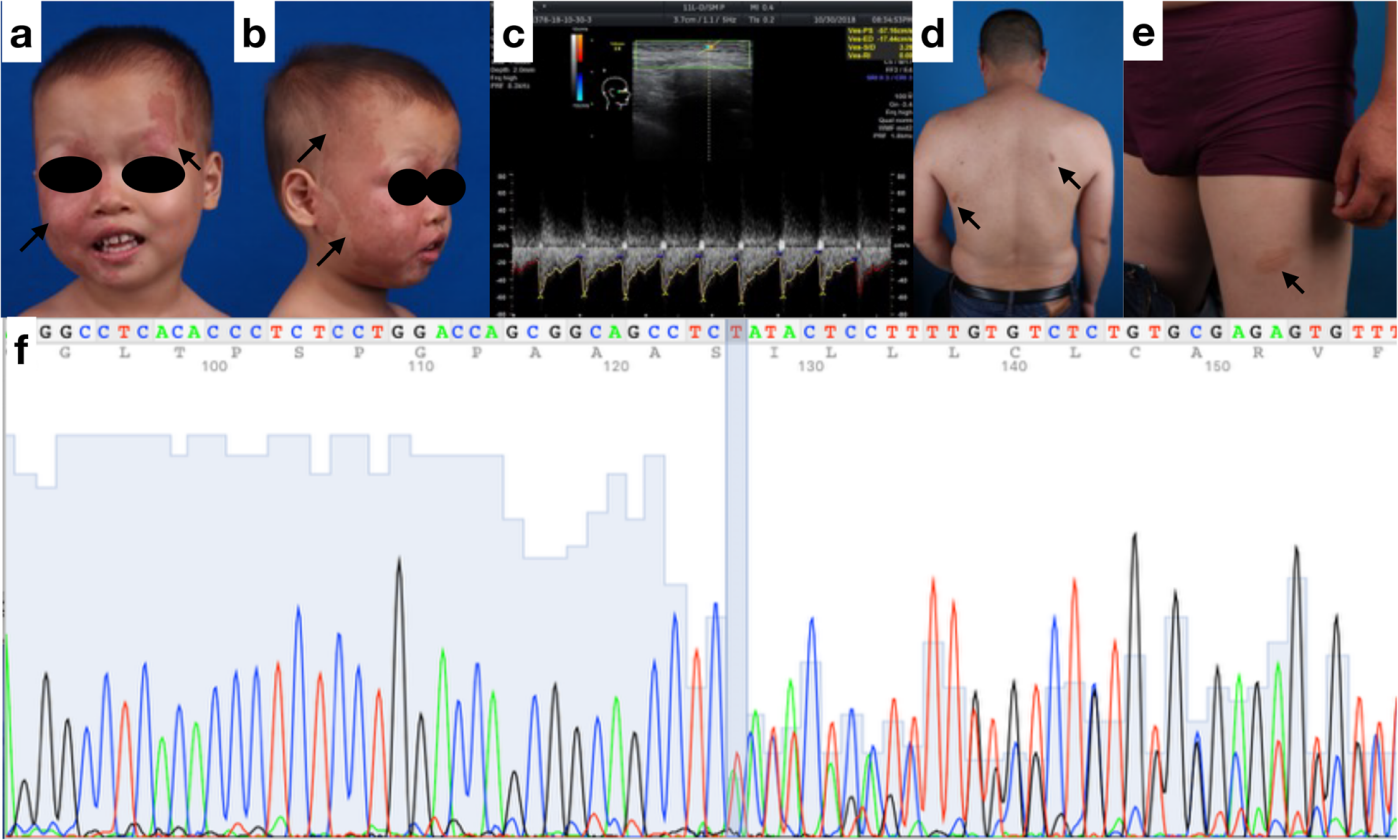
**a**-**b** Clinical manifestation of the patient. **c** Ultrasound scan of the patient in the facial lesions. **d** lesions in the back of proband’s father. **e** lesions in the leg of proband’s father. **f** Sanger sequence of germline mutation of EPHB4(7:100402906 AGT > A)

A 4-year-old boy with facial erythema visited our clinic with his parents. He was born at 35 weeks to a 41-year-old mother via eutocia. The lesions appeared darker on his face 2 weeks after birth. The red lesions were located in the V1 region of his left face, the V2 and V3 regions on his right side, and the nasal back (Fig. 1a-b). Half of the lip was involved, but there were no lesions on the gingiva. The lesions had become progressively darker and larger, as reported by his parents. We were told that the boy was diagnosed with port-wine stains, received medical treatment for Pulse dye laser (PDL) with no effect and underwent regular clinical follow-ups for Sturge-Weber syndrome (SWS) as a precaution.

Family history showed that the child’s father presented two erythemas on his back and leg (Fig. 1d-e).

During physical examination, the skin temperature of the lesion was found to be slightly higher than that of the adjacent normal area. The child was examined with no indications of glaucoma or potential seizures.

An ultrasound scan showed a hyperechoic signal with a peak artery speed of 37–54 cm/s (Fig. 1c).

To identify genetic changes, we performed a minimally invasive needle biopsy (D = 2 mm, all layer biopsy) under local anesthesia (Penles and 1% xylocaine). Targeted next-generation sequencing (NGS) was then performed on blood specimens from the peripheral vein as well as tissue specimens. The targeted gene panel for NGS was designed according to the ISSVA classification [10].

### Next-generation sequencing (NGS)

To target the exons as well as the exon/intron boundaries of EPHB4, a series of RNA capture baits were designed. SureSelect XT kit reagents (Agilent Technologies, Santa Clara, CA) were used for the Illumina adapters. Quantitative PCR (KAPA Biosystems, Wilmington, MA) was used to verify the concentration of the indexed sample, and the sample was then sequenced on a MiSeq instrument (Illumina, San Diego, CA), generating 2150 paired-end reads. The variants were analyzed by using the Integrative Genomic Viewer.

### Sanger sequencing

The EPHB4 gene was PCR-amplified to track 7:100402906 AGT > A, c.2714_2715delAC, p.His905fs in the blood and tissue samples. The primer sequences that were used are available upon request. The amplicon fragments were sequenced bidirectionally (forward and reverse) with the M13 primer by using the Big Dye1 Terminator v3.1 cycle sequencing kit and an ABI 3730 DNA Analyzer (Life Technologies, Carlsbad, CA). The target sequences were compared to the EPHB4 reference sequence using Mutation Surveyor (SoftGenetics, State College, PA).

## Result

A genetic study revealed that the patient and his father harbored a germline mutation in EPHB4 (p. His905fs). NGS analysis showed this mutation was detected with a mutation frequency of 47 and 41.5% the patient’s and the father’s blood sample. The depth of coverage for this EPHB4 mutation was 6121x in the patient’s blood sample (Fig. 1f). In contrast to previous diagnoses, based on the family history of erythema, the results of a physical examination and ultrasound raising potential fast-flow lesions, and a genetic study revealing a germline EPHB4 mutation, we diagnosed the child with capillary malformation-arteriovenous malformation type 2 (CM-AVM2).

## Discussion

CMs are composed of dilated capillary-like channels in the dermis [11]. Although previous studies have reported heredity components of CMs with an inheritance rate of 8–22% [12], a CM is generally not considered to be a familial condition. Clinically, CMs are characterized as reddish-purple lesions with flat surfaces, most of which occur in the head and neck area. Approximately 26% of PWSs located in the distribution area of the ophthalmic branch of the trigeminal nerve are associated with Sturge-Weber syndrome (SWS), a neurocutaneous disorder. SWS is characterized by facial PWSs, venous capillary anomalies of leptomeninges in the brain and eyes, seizures, stroke, glaucoma, and intellectual disability [13]. In 2013, Shirley et al. found that PWSs and SWS were caused by somatic GNAQ (R183Q)-activating mutations, inducing the activation of the mitogen-activated protein kinase (MAPK)/extracellular signal-regulated kinase (ERK) signaling pathway and leading to angiogenesis and increased migratory behavior in vitro [14].

The distinct phenotype consisting of multiple small, round-to-oval CMs with surrounding pale halos was associated with germline mutations of RASA-1 and designated capillary malformation-arteriovenous malformation (CM-AVM) in 2003 [4]. A “second hit” hypothesis was proposed as the pathomechanism of CM-AVM [4, 15]. Later, in 2017, a novel germline EPHB4 mutation was detected in RASA-1-negative CM-AVMs, mimicking RASA1-related CM-AVM1 and HHT [8]. These EPHB4-related CM-AVMs were referred to as CM-AVM type 2.

According to the literature, in addition to multiple sites of erythema, CM-AVM patients exhibit an increased risk of fast-flow lesions such as arteriovenous malformation (AVM) or arteriovenous fistula (AVF), or even hypertrophic syndromes such as Sturge-Weber syndrome (SWS) and Parker-Weber syndrome (PKWS) [16, 17].

According to the clinical aspects of the case described herein, the congenital unilateral and segmental facial erythema was distributed along the ophthalmic branch of the trigeminal nerve, located in the V1 region of his left face and the V2 and V3 regions on his right side (Fig. 1a-b). Dermatologists or primary care providers may have diagnosed his lesions as port-wine stains and sent the boy for a glaucoma examination and a neurologic examination for potential seizure attacks. Physical examination showed a higher temperature of the red lesions than the adjacent or normal tissue, which may be suggestive of fast-flow lesions. An ultrasound scan confirmed our concern, where the signal of the fast-flow lesions revealed a peak artery speed of 37–54 cm/s. Family history analysis showed the existence of hereditary multifocal red lesions. A genetic study confirmed our concern regarding the molecular aspect of the condition. We diagnosed the child with capillary malformation-arteriovenous malformation type 2. With the potential for AVM attacks and considering the potential harm caused by radiation, digital radiography was not performed on the patient. We performed regular clinical follow-up in the child as a precaution related to a potential AVM attack. Bleomycin injection is considered as a medical method of treating AVM at an early stage.

This study emphasizes the limitations of a single clinical diagnosis and the importance of molecular diagnosis. As dermatologists, we can easily overlook a potential AVM under erythema lesions and choose PDL or PDT for treatment, leading to a missed diagnosis of CM-AVM and early treatment of the underlying AVM. The potential life-threating.

complications of AVM, including heart failure, hemorrhage and seizures, cannot be neglected [4, 16, 18]. We recommend the use of NGS together with a careful review of the medical history, physical examination results and ultrasound scan analysis data to facilitate the evaluation of the potential risk of fast-flow lesions. Patients should have undergo clinical follow-ups, be made aware of the potential fast-flow lesion, and receive appropriate initial treatment.

## Conclusion

Herein, we report one male patient with facial erythema who was initially thought to have PWSs because of the unilateral and segmental distribution of his red facial lesions but was later diagnosed with CM-AVM2 syndrome based on a germline EPHB4 mutation. It would be valuable to obtain clinical information as well as the relevant molecular diagnosis. Such information would help us to understand the differential diagnosis of isolated CMs and CM-AVMs and to evaluate the potential risk of fast-flow lesions underlying erythema.

## Data Availability

All data used during the study appear in the submitted article.

## Abbreviations

CM-AVM: Capillary malformation-arteriovenous malformation
CMs: Capillary malformations
HHT: Hereditary hemorrhagic telangiectasia
AVF: Arteriovenous fistula
CTA: Computer tomography angiography
AVM: Arteriovenous malformation
PKWS: Parker-Weber syndrome
PWSs: Port-wine stains
SWS: Sturge-Weber syndrome

## References

[1] Jacobs AH, Walton RG. The incidence of birthmarks in the neonate. Pediatrics. 1976;58(2):218–22. 951136

[2] Morelli JG (1989). Vascular birthmarks: Hemangiomas and malformations. Br J Plast Surg.

[3] Wassef M, Blei F, Adams D (2015). Vascular anomalies classification: recommendations from the International Society for the Study of vascular anomalies. Pediatrics..

[4] Eerola I, Boon LM, Mulliken JB (2003). Capillary Malformation–Arteriovenous Malformation, a New Clinical and Genetic Disorder Caused by RASA1 Mutations. Am J Hum Genet.

[5] Wooderchak-Donahue WL, Johnson P, McDonald J (2018). Expanding the clinical and molecular findings in RASA1 capillary malformation-arteriovenous malformation. Eur J Hum Genet.

[6] Wooderchak-Donahue WL, Akay G, Whitehead K, Briggs E, Stevenson DA, O'Fallon B, Velinder M, Farrell A, Shen W, Bedoukian E, Skrabann CM, Antaya RJ, Henderson K, Pollak J, Treat J, Day R, Jacher JE, Hannibal M, Bontempo K, Marth G, Bayrak-Toydemir P, McDonald J. Phenotype of CM-AVM2 caused by variants in EPHB4: how much overlap with hereditary hemorrhagic telangiectasia (HHT)? Genet Med. 10.1038/s41436-019-0443-z 30760892

[7] Cai R, Liu F, Hua C (2018). A novel RASA1 mutation causing capillary malformation-arteriovenous malformation (CM-AVM): the first genetic clinical report in East Asia. Hereditas..

[8] Amyere M, Revencu N, Helaers R (2017). Germline loss-of-function mutations in EPHB4 cause a second form of capillary malformation-arteriovenous malformation (CM-AVM2) deregulating RAS-MAPK signaling. Circulation..

[9] Edwards LR, Blechman AB, Zlotoff B. RASA1 mutation in a family with capillary malformation–arteriovenous malformation syndrome: A discussion of the differential diagnosis. Pediatr Dermatol. 2018;35(1):e9–e12. 10.1111/pde.13332. Epub 2017 Nov 9. 10.1111/pde.13332 29120072

[10] Blei F (2017). ISSVA Classification of Vascular Anomalies.

[11] Barsky SH, Rosen S, Geer DE, Noe JM. The Nature and Evolution of Port Wine Stains: A Computer-assisted Study. J Investig Dermatol. 1980;74(3):154–7. 10.1111/1523-1747.ep12535052 7359006

[12] Rubin AT, Lauritzen E, Ljunggren B, Revencu N, Vikkula M, Svensson Å. Heredity of port-wine stains: Investigation of families without a RASA1 mutation. 10.3109/14764172.2015.1007060 PMC4975081 25602354

[13] Comi AM (2007). Update on Sturge-weber syndrome: diagnosis, treatment, quantitative measures, and controversies. Lymphat Res Biol.

[14] Shirley MD, Tang H, Gallione CJ (2013). Sturge-weber syndrome and port-wine stains caused by somatic mutation in GNAQ. N Engl J Med.

[15] Revencu N, Boon LM, Mendola A (2013). RASA1 mutations and associated phenotypes in 68 families with capillary malformation-arteriovenous malformation. Hum Mutat.

[16] Veerse F, Thepaut JN (2010). Parkes weber syndrome, vein of Galen aneurysmal malformation, and other fast-flow vascular anomalies are caused by RASA1 mutations &dagger. Hum Mutat.

[17] Hershkovitz D, Bercovich D, Sprecher E, Lapidot M. RASA1 mutations may cause hereditary capillary malformations without arteriovenous malformations. Br J Dermatol. 2008;158(5):1035–40. 10.1111/j.1365-2133.2008.08493.x. Epub 2008 Mar 20. 10.1111/j.1365-2133.2008.08493.x 18363760

[18] Orme CM, Boyden LM, Choate KA, Antaya RJ, King BA. Capillary Malformation-Arteriovenous Malformation Syndrome: Review of the Literature, Proposed Diagnostic Criteria, and Recommendations for Management. Pediatr Dermatol. 2013;30(4):409–15. 10.1111/pde.12112 23662773

